# Characterization of High Purity Silicides

**DOI:** 10.6028/jres.093.029

**Published:** 1988-06-01

**Authors:** Purneshwar Seegopaul, Maria C. L. Williams

**Affiliations:** Materials Research Corporation, Orangeburg, NY 10962

The semiconductor industry has produced some exciting and dramatic advances in technology whose impact has been felt universally. Integration of an increasing number of devices on single microchips has necessitated the use of specialized and highly pure materials. The interconnect materials pose unique difficulties as resistivity limitations are reached with the traditional aluminum and polysilicon. Silicides are now being utilized to overcome some of these limitations in interconnect technology. These silicides are basically compounds of silicon with the refractory element of choice in varying stoichiometries that fit the need of the process and metallization sequence. Some common silicides are compounds of silicon and tantalum, tungsten and molybdenum. Silicides are produced by melting silicon with the refractory element of interest.

Characterization of high purity silicides involves both physical and chemical properties. Table 1 lists some of the parameters involved in characterization of these materials. This discussion will focus on the chemical properties of purity and stoichiometry. The silicon to metal ratio is critical to the needs of the application and impurities are incompatible with yield, quality, and reliability of the devices.

Table 2 describes a general strategy in generating the purity and stoichiometry of the silicides. Both “wet” solution-based and “solid”-based methods are exploited for optimum analytical yield and reliability. The techniques include spectroscopic methods for compositional and impurity determination and “LECO” combustion methods for gases. The spectroscopic techniques include atomic absorption, atomic emission, x-ray and mass spectroscopy.

Sampling and dissolution of the material for the solution methods are critical. Powdered samples are mixed and blended to ensure homogeneity, while large samples are reduced to small pieces followed by random sampling for dissolution. Dissolution of the silicides introduces the need for special precaution to prevent loss of silicon through volatilization. This is obviously critical in the compositional analysis. Methods for dissolution are generally based on acid or fusion procedures. The fusion methods use sodium peroxide/sodium carbonate mixtures while the acidic methods utilize a mixture of hydrofluoric acid, nitric acid and water. This work is based on a rapid acidic dissolution method at room temperature. Previous acid methods were very lengthy and involved used of a liquid nitrogen blanket for cooling. The accuracy and precision of this technique were studied and table 3 shows correlation data for % silicon determination by several techniques. The excellent agreement demonstrates the validity of the rapid acidic dissolution without loss of silicon. The % silicon is routinely measured by atomic absorption techniques with a wavelength of 251.6 nm in a nitrous oxide/acetylene flame. The data in table 3 clearly show that any of these methods may be used for this analysis.

As discussed earlier, a combination of analytical techniques are used for determining the impurities in the silicides. These impurities are generally metallics, gases and interstitial elements. The determination of metallics is accomplished by the spectroscopic methods while the gases are measured by combustion type techniques.

The determination of metallics involve sampling, etching to remove surface contamination and dissolution to a 2.5 % w/w solution for analysis. Matrix matching is critical for accurate and precise analysis. All standards are, therefore, properly matrix matched to reflect the stoichiometry of the silicide being analyzed. Incorrect matrix matching results in the suppression or enhancement of the signals used to generate final analytical values. Reference and duplicate samples are routinely analyzed to ensure accuracy and precision. The routine methods consist of ICP and DCP plasma emission spectroscopy and atomic absorption spectroscopy. The ICP spectrometer is equipped with a holographic grating while the DCP spectrometer has an Echelle grating.

Table 4 shows some correlation data of impurities determined in a moly silicide by different techniques. Most of the elements showed excellent agreement between the methods used to generate the data. Copper and chromium gave poor recovery type data in the plasma emission techniques and are analyzed by atomic absorption methods. Interlaboratory studies of silicide analysis were also conducted and the results again revealed excellent correlation.

This discussion highlighted the importance of silicides in the semiconductor industry and the need for accurate and precise analysis. Purity and composition can be readily determined by both atomic absorption and plasma emission techniques.

## Figures and Tables

**Table 1:** Characterization of high purity silicides

Physical properties
Resistivity
Density
Homogeneity
Grain size
Microstructure
Mechanical strength
Temperature profiles
Chemical properties
Etchability
Purity
Stoichiometry
Thin film properties
Physical
Chemical

**Table 2:** Analytical chemistry of silicides

Analytical parameters
Stoichiometry—Composition
Purity —Impurity content
Techniques
“Wet” solution-based methods
a. Atomic absorption spectroscopy
Flame/graphite furnace
b. Plasma emission spectroscopy
Direct current plasma (DCP)
Inductively coupled plasma (ICP)
“Solid”-based methods
a. Energy dispersive x-ray spectroscopy
b. Mass spectroscopy
Glow discharge
Spark source
c. Combustion “LECO” methods

**Table 3:** Correlation data of % Si in silicides

Sample	Atomic absorption		Atomic emission			
			DCP		ICP	
	Duplicate	%Rec	Duplicate	%Rec	Duplicate	%Rec
MoSi_2.0_	36.88	99.5	37.69	99.2	36.84	99.9
	37.00		36.76		36.59	
MoSi_1.7_	32.60	99.2	32.80	99.1	32.60	98.5
	33.60		32.60		32.40	
TaSi_2.5_	27.98	99.0	28.60	98.4	29.00	99.2
	27.70		27.90		28.50	
W_5_Si_3_	8.20	99.0	8.40	98.9	8.43	98.8
	8.30		8.32		8.42	

**Table 4:** Correlation results of impurities

Analyte	AAS	DCP	ICP	MS

	(ppm)			
Al	<1	<1	0.8	1
Ca	<1	0.9	1.1	0.7
Cu	6	20	14	5
Cr	4	14	25	3
Ni	9	6	6	3
Co	3	<1	<1	0.04
Mg	<1	3	<1	1
Fe	18	17	20	15
Mn	<1	<1	<1	0.1

